# Educational supervisor’s perceptions of their role in supporting residents’ learning: a qualitative study

**DOI:** 10.5116/ijme.6544.cf18

**Published:** 2023-11-20

**Authors:** Cecilie N. Birkeli, Karin Isaksson Rø, Lisbeth Rustad, Monika Kvernenes

**Affiliations:** 1 Institute for Studies of the Medical Profession, Oslo, Norway; 2 Center for Medical Education and Department of Clinical Medicine, Faculty of Medicine, University of Bergen, Norway

**Keywords:** Educational supervision, supervisory roles, residency training, workplace learning, postgraduate medical education

## Abstract

**Objectives:**

During the past decade, educational supervision (ES) has gained popularity as a key support mechanism in residents’ training. However, few studies have mapped physicians’ understanding of their roles as educational supervisors. This study aims to explore how supervisors experience this role and how they approach providing support to residents.

**Methods:**

We employed qualitative methodology and conducted semi-structured interviews with 13 senior hospital physicians regarding their experiences as educational supervisors. Participants were recruited via e-mail and snowball sampling. Interview transcripts were analysed using inductive systematic text condensation following a four-step procedure: (i) total impression (ii) identifying and sorting meaning units (iii) condensation from code to meaning (iv) synthesising condensation from description and concepts.

**Results:**

Our analysis yielded four main themes. We found that while ES was considered important in theory, its purpose appeared unclear in everyday practise. Second, ES was associated with filling multiple and sometimes contradictory expectations. Third, establishing a good relationship between residents and supervisors was considered critical for effective ES. Finally, being a supervisor was described as experiencing a personal cost in the absence of support and resources.

**Conclusions:**

The many roles embedded in attending physicians' understanding of ES indicate a wide definition of the supervisory role. Supervisors contribute to residents' training by ensuring educational quality while upholding quality in patient care. Educational supervisors considered themselves to be vital sources of support for residents, but found their role unclear in everyday practice. They strove to manage different expectations resulting from lack of supportive organisational structures.

## Introduction

Supervisors play a vital role in residents’ training, and previous research has shown that supervision in clinical training contributes to better learning outcomes and improvement in patient treatment.^1^^-^^3^ In recent years, many residency programs have developed new standards for postgraduate training, with an increased emphasis on educational supervision (ES). ES,^4^ can be defined as: ‘regular supervision taking place in the context of recognised training, in order to establish learning needs and review progress’. In contrast to traditional clinical supervision (CS), which is commonly associated with the apprentice model of residency education, ES is characterised by regular reflection-based, one-to-one supervisory meetings organised as conversations about residents’ experiences, learning needs, and progression.^5^ It is recommended that ES occurs in longitudinal relationships, with meetings scheduled at regular intervals. In addition, residents have clinical supervisors with whom they can consult in their daily work, requiring a close connection between supervisor and learners, although clinical supervisors can shift daily.^4^

A prevailing problem in the literature is the poor delineation of the various supervisory roles and support functions that attending physicians may have towards their residents.^4^ Supervisory functions have been found to incorporate many tasks, such as clinical, educational, personal, and interpersonal supervision.^4^^-^^6^ Consequently, many physicians struggle to identify with the multiple educational roles that they are expected to fill. Although attending physicians are considered important to resident learning, few studies have explored how they perceive and experience their role as educational supervisor.^6^

From an educational perspective, hospital workplaces are seldom optimally organised for resident learning because training activities can often be squeezed in between the treatment of patients and performing job-related tasks.^7^^,^^8^ With healthcare service demands as the backdrop, supervisors are expected to find opportunities to aid residents in their educational progress. However, it can be claimed that imprecise and negatively focused descriptions of workplaces, such as informal and unstructured descriptions of learning, do little to broaden understanding or improve the status of the workplace as a learning space. Providing an alternative approach, Billett^9^^,^^10^ introduces the term workplace affordances. Workplace affordances identify factors that can contribute to opportunities for learning, and the ways in which individuals choose to engage in work activities. Workplace readiness in providing opportunities for individuals to engage in work activities and have access to support are important determinants of workplace learning quality.

Little is known about how supervisors perceive their role in supporting resident learning or how they conduct ES.^6^^,^^11^ Their perspectives can, however, provide valuable insight that will contribute to inform and improve ES practices. This study explores how supervisors experience their own roles in ES through their own descriptions of how it is conducted at work. Using a qualitative approach, this study investigates supervisors’ experiences with ES through individual interviews.

The research question is:

How do supervisors reflect upon the importance of educational supervision and consequently their own role in the residents´ training in hospitals. What responsibility do they take during the residents´ training?

## Methods

### Research context

In Norway, a competency-based model (CBME) of residency training was introduced in 2017 for internship and, in 2019, for residents, with a three-part specialisation pathway for residents.^12^ Health authorities initiated major revisions that included changes in responsibilities, roles, and assessment regimes. Compulsory service times, fixed numbers of completed procedures, and compulsory courses as measurements of competence were partly replaced by learning objectives, and the focus on supervision and continuous assessment was sharpened. The new guidelines state that residents are to be appointed a permanent supervisor who, ideally, will follow the resident throughout his/her entire residency with regular meetings of 45-60 minutes every four weeks. Supervisors are expected to initiate the first supervisory meeting no later than three weeks after the candidate has started.^13^

### Study design

We conducted a qualitative explorative study using individual semi-structured interviews enabling us to explore supervisors’ experience and reflection on ES in-depth.^14^ Interviews were deemed to be an appropriate method to access participants´ perspectives and relevant experiences, in this case, to gain insight into how they experienced ES.^15^

### Participants

Participants in this study were senior hospital consultants who had supervised one or more residents throughout their specialist training. Recruitment was initially done by an open e-mail invitation to a sample of 200 consultants in internal medicine and surgery based on a random extract from the Norwegian Medical Association’s membership register. Only five participants responded, forcing us to change our recruitment strategies. Using the first five participants as a starting point, we recruited the remaining participants by snowballing.^16^ We ensured an even gender distribution and, additionally, recruited supervisors from geographically diverse locations. Our sample consisted of 13 supervisors, six female and seven male, from nine hospitals across the country. Six were internal medical specialists, six were surgical specialists, and one was a specialist in anaesthesiology. Thus, we included specialist from the large groups of internal medicine and surgical specialties. We did not include family medicine or psychiatry, as we considered these specialties to have supervisory traditions distinct from those of somatic hospital-based specialties.

Before conducting the interviews, participants received oral and written information about the study, including information about voluntary participation and data management. All queries were clarified. After the participants provided informed written consent, semi-structured individual interviews were conducted in a preferred and convenient location for the participants,^17^ who could choose between a physical or a digital meeting. We emphasised participants’ ability to speak freely, treating their data confidentially. This study was submitted for assessment and was exempted from approval by REK (Regional committee for medical and health research ethics) ref.nr 2018/283 B. However, it was registered in System for Risk and compliance - Processing data in research and student projects at University of Bergen, with ID: F1508.

### Data collection

We conducted semi-structured interviews using a set of predetermined, open-ended questions to collect data.^17^ This approach allowed participants to talk about supervision freely and introduce descriptions as they saw fit. The interview guide was piloted and revised before data collection commenced (Appendix). Three supervisors chose to be interviewed in physical meetings, whereas the remaining ten chose digital interviews conducted via Teams. All interviews were audiotaped, transcribed verbatim, and anonymised before being shared with the research team. The interviews were conducted from October 2021 to May 2022 and lasted for 30-60 minutes.

### Data analysis

Data were analysed using systematic text condensation (STC).^18^ This method is a descriptive and exploratory form of thematic cross-case analysis, and presents a pragmatic approach consisting of a four-step procedure: (i) gaining a total impression of the material and selection of preliminary themes, (ii) identifying and sorting meaning units and developing code groups, (iii) condensation from code to meaning, and (iv) synthesis condensation to descriptions and concepts. Based on these initial impressions, a codebook was developed. The first author (CNB) organised all transcripts in line with the codebook using NVIVO and Word software. We established and identified quotes that illustrated the meaning and contents of the subgroups. Finally, we synthesised condensations for descriptions and concepts.

### Trustworthiness and rigor

In this study, we drew on our experiences in various pedagogical, health science, and medical practices. Two authors (KIR and LR) are experienced medical doctors with first-hand knowledge of residency supervision. The interviewing author (CNB) has extensive experience in evaluating residents’ training in hospitals. This was considered an important prerequisite for facilitating conversations about supervision and gaining participants’ trust.^19^ MK has a professional background in higher education, and a particular interest in faculty development. The first author (CNB) conducted all 13 interviews, transcribed five of the interviews and analysed all interviews in step (i). The other three authors (KIR, LR, and MK) read five interviews each. This ensured a solid foundation for discussing the overall impressions and preliminary themes. During this process, the author group held regular meetings to discuss and adjust the codes. This process was iterative, and we repeatedly consulted the material during the revision and adjustment of the codes. Throughout the process, the research question guided the adjustment and revision of the codes.

We attempted to provide a nuanced understanding of the material and repeatedly discussed how our previous experiences, understanding of the material, and possible biases could have affected the analysis. CNB drafted the manuscript with help from the other authors. To ensure internal validity and to increase credibility and confirmability,^20^ all the participants were offered a citation check of the results to ensure that the data were recognisable to them.^21^

## Results

Our analyses yielded four themes, all of which represented key aspects of the supervisory role. These themes are elaborated on below.

### Educational supervision is important in theory, but unclear in everyday practice

Supervisors asserted ES as an important feature of specialist training, involving regular one-to-one conversations between residents and a more experienced consultant who was given a formal supervisory role. The ES structure was organised differently between departments and hospitals. Some supervisors described ES as a conversation with regular appointments, or as a program that was intended to be implemented regularly, once a month. The meetings typically addressed career management, specific situations that residents had experienced, ethical dilemmas, and work-life balance. Furthermore, supervisors described how they aimed to provide support to residents in providing quality care, attending to patient safety, collaborating in teams, handling their own and others’ expectations of the physician’s role, and dealing with time management and job stress. One participant described a recent ES session as follows.

“..the last time we had ES, my candidate told me about her last shift, where she had 112 calls that she had to answer (...) with inquiries about all sorts of things. And like, how do you deal with that? What can you do with the large volume of work – you may not be able to do anything about it, but how do you weather the storm.” (Interview 9, Female, internal medicine)

While the supervisors recognised the theoretical distinction between ES and CS and acknowledged both support structures as important, the delineation between their roles as educational supervisors and clinical supervisors in daily workplace interactions seemed blurred. Several supervisors pointed out that the delineations offered in faculty development courses on how to supervise came across as idealistic and theoretical, but unproductive in everyday clinical work. The dominant view was that day-to-day CS was considered more important than scheduled supervisory conversations (ES). One participant described this as follows: 

“Well... the formal supervisory role is very limited. It's like... you find a suitable time and have a half an hour/45 min. talk... whenever we can find the time, depending on what that candidate wants really. (…) and I really think the value of those meetings, the formal educational supervisory meetings, I think at least the way it works with us, is low. It's the day-to-day supervision, the master-apprentice supervision that you get from the people you work with that is much more important.”  (Interview1, Male internal medicine)

A recurring concern was that the supervisors did not consider supervisory conversations with residents who did not have problems to be time well spent. However, they presumed that ES would become more relevant if they were to have residents who needed closer follow-up.

“So, it's okay that we are expected to have supervisory meetings. In the new system, we are supposed to have frequent meetings, but I am not convinced how... or what you need them for. But I guess there may be people who have more psycho-social problems or things like that, which means that you need some follow-up, but, as a formal part of the residents' education, I don't think you need..., perhaps you need three or four conversations a year, just to catch up on if there are any issues.” (Interview 8, Female, surgeon)

### Supervisors balancing multiple expectations

ES was perceived to be the responsibility and duty of senior hospital physicians as a part of their job. Supervisors reported that they felt obliged to provide professional and personal support to residents and, at the same time, to assess their performance and steer residents on alternative career paths if they were deemed unfit to pursue their chosen speciality. Furthermore, they claimed that the role included ensuring that residents had access to appropriate learning opportunities, including teaching, clinical training, and ES. Finally, being a role model was described as an important part of their supervisory tasks.

The supervisors considered themselves to be support persons who contributed with knowledge, experience, advice, expertise, and professional proficiency to residents´ training. They recognised that ES should be based on residents’ needs and reflections. Sometimes, this included being a supportive colleague when residents experienced difficulties, and an advocate for management or the community in general when the residents needed someone to argue their case for them. Several supervisors explained that they used ES meetings to boost residents’ confidence. Guiding residents towards job proficiency was considered a goal. However, management of the work-home balance would, at times, require attention, leaving supervisors with the challenge of finding an appropriate balance between personal and professional support during ES meetings. One participant described this as follows:

“(...) And then the conversation turned to how life was in general because she's had a bit of a tough time on the home front with a sick husband. So, I feel like I've been..., yes, I actually feel like I've been a pretty important supporter for her through that. Not so much professionally, because she's done really well there, but in terms of that personal level.”  (Interview 10, Female, internal medicine)

Supervisors also felt responsible for meeting expectations in assessing residents’ performance, which was emphasised as being important in determining whether residents had reached the expected professional level. This is particularly important from a patient safety perspective. However, supervisors reported that they found it challenging not to let assessments dominate supervisory meetings. One supervisor claimed that as a result of the increased focus on assessment attached to the introduction of CBME, residents had become more passive:

“I don't think it was necessary to go as far as one has done now, because now the training of surgeons has been turned into almost ... a checkbox, a flowchart where you must fill in what you have done ... and then you just sum it up and you get a new specialist out in the other end. And it doesn't quite work that way. Residents are at risk of becoming clients more than colleagues (...) It's at such an incredible level of detail that it will..., it is almost counterproductive, because it is so extensive, that no one has the overview anymore.” (Interview 3, Male, surgeon)

### Establishing a good relationship between resident and supervisor is key to well-functioning ES

Most supervisors asserted that they tried to function as trusted allies for residents, so that learners would feel comfortable seeking advice, receiving feedback, talking about dilemmas, and raising sensitive issues in meetings. Having been in their situation, supervisors also acknowledged the importance of showing empathy toward residents and their learning situation.

“It is hard to be a doctor in the specialist health service, there is a lot we experience, a lot that can be painful and difficult and I try to convey that that’s the way it is… because everyone has done stupid things that weren’t that good. It's a lot like that, I feel… And it must be, we're only human.” (Interview 4, Female, surgeon)

According to supervisors, establishing a trusting and caring relationship requires effective communication skills and a good match of personalities.

“It is obvious that in (educational) supervision very much is person-dependent and having a person that you for some reason just do not click with… we are very different people and sometimes it is uncomplicated and other times, perhaps you just don’t communicate well, it can be difficult. If I get a resident who somehow does not match personality-wise or communication-wise (with me), it can be very difficult indeed.” (Interview 2, Male, surgeon)

The frequency and type of contact between supervisors and residents in the workplace were found to affect their relationships. Here, two main patterns emerged. One relationship pattern was characterised by frequent contact between supervisors and residents through daily interactions in the workplace. Proximity allowed supervisors to monitor the residents’ work and progress, and feedback was based on direct observations from the supervisors. This relationship pattern was particularly evident within procedure-heavy disciplines, such as surgery, and in departments with little rotation among staff. ES in the form of scheduled conversations did not necessarily take place regularly, but was scheduled when deemed necessary; for instance, when the resident wanted to discuss particular incidents, or if clinical supervision was not optimal. One participant described how working side by side with a resident minimised the need for ES:

“And then you sit in ES sessions wondering what we are really going to go through in these meetings when things are working fine, right. And then maybe with some residents where things have not worked out that well, it is good to have an (educational) supervisor as well, right.”  (Interview 13, Female, internal medicine)

The other relationship pattern identified involved supervisors who were more disconnected from the residents’ daily work. These supervisors had few opportunities to observe candidates in daily practice. This distance was often a result of departments organised in outpatient and inpatient clinics and frequently rotating work shifts, where residents would rotate between posts. The lack of proximity between supervisors and residents challenged the relationship because many supervisors felt that they lacked an overview of the residents’ level of knowledge. Hence, they found it challenging to provide feedback on situations in which residents experienced something difficult, such as being left alone during difficult patient conversations, or problematic incidents that occurred in the working team with which the resident was involved.

“I find it difficult if my resident talk about problematic things that the person is experiencing and then I hear the same thing presented, i.e., the same situation presented by someone else with... for example a completely different angle or a completely different experience of the situation.” (Interview 10, Female, internal medicine)

### Being a supervisor has a personal cost in absence of support and organisational resources

The supervisors found that contributing to the education of specialists was a meaningful and important aspect of their work. However, they claimed that it was difficult to compensate for the absence of the basic resources and structures needed to do the job well. Most were self-taught supervisors and reported having few guidelines or instructions on how to supervise. Hence, their approach to and understanding of ES was formed by their own experiences of being supervised as residents. Furthermore, the tasks and expectations that come with the role were tacitly communicated through the priority it was given by colleagues and leadership. Some had attended formal supervisory courses, however, not all found the training to be clarifying or relevant to their job situations. One participant expressed the following:

“It’s just like someone has created a course for how it should be. (...) And I never feel like they can concretise it. I've asked them many times "what do you really mean", so I still feel the supervisor role, that's what I really thought at the start, and I still feel that the supervisor role is a bit unclear, for me. Has been unclear throughout my education; (...) And it annoys me a little bit.” (Interview 13, Female, internal medicine)

Finding time for dedicated ES meetings was described as challenging, and most supervisors were left to find that time within busy clinical schedules. When prioritising ES, they had to compensate for the time spent by catching up with the clinical work or by supervising after working hours. Because of this lack of organisational support, regularly scheduled ES was often less prioritised, and was instead scheduled on an ad hoc basis, when the opportunity arose. Although supervisors reported that there was no scheduled time for ES in their work plans, residents usually had scheduled time for ES in their educational plans. One supervisor described ES as having a personal cost because he sacrificed his own free time to ensure that ES was offered:

“If you take that afternoon off (ad: from the clinic to provide educational supervision), then someone else will have to work for you. And why do I have to negotiate that? If the hospital wants to have residents and if the hospital wants to have a better education, then it must come from the top (…) The more involved I am in supervision and mentoring, the more I lose in terms of having a relaxed life. For now, it's going well, but I wonder why that cost must be personal (…) The question is time, can they give me extra time.”  (Interview 5, Male, anaesthesiology)

Overall, participants expressed lack of opportunities and collegial culture to discuss and share their experiences as supervisors. However, some said that they met with fellow supervisor bi-annually in so-called ‘assessment panel meetings’ to discuss the progression of their residents. Although these meetings represented a forum in which supervisors could meet and share their experiences, they also commented that focusing on assessment could be potentially problematic. It was argued that residents might see the assessment as being high stakes, and that they should be allowed to be present in meetings where their progression was discussed. One supervisor referred to assessment panel meetings as a promising idea, but one which could potentially do harm:

“The intention is good, but my biggest concern is ethical... Yes, what I struggle with the most, about that evaluation collegium, is that the most important person is not in the room. The resident. I don't like that people who have more or less a good basis for speaking out have an arena where you can... yes... do potentially great damage to peoples' names and reputations within a department.”  (Interview 11, Male, internal medicine)

Most supervisors described lack of organisational resources to conduct ES regularly. Some of them claimed that residents were afraid to speak up about time pressure and heavy workloads, for fear of losing their temporary positions as junior doctors during training. Supervisors compensated for this absence of support and organisational structures by taking personal responsibility in ensuring the supervision and follow-up of residents during their education. Several created their own schemes and tools that they believed strengthened and structured their supervision. At the same time, the supervisors also expressed uncertainty as to whether the strategies or tools they used were good enough. One supervisor expressed that she felt her authority to define ‘best practice’ as daunting:

“Yes, it is, because as I said, communication is very, very important to us, and then there is communication with both patient and often relatives. It can be quite demanding because there are so many ways to react. And when you …have a conversation like that, that's it, then it's like my way of doing it is kind of “the way it’s done”. And I don't know if my way is the right way or the terrible way. I don't get any correction on that either if you understand.” (Interview 10, Female, internal medicine)

## Discussion

Supervisors play a significant role in residents’ training and in the implementation of the curriculum in the workplace.^22^^,^^23^ Our study identified four themes demonstrating that supervisors contribute to residents’ learning on multiple levels by ensuring education and upholding quality in patient care. We discovered that supervisors have applied a broad definition of supervision and supervisory roles, which incorporates many, sometimes conflicting responsibilities. Supervisors in our study acknowledged the theoretical distinction between ES and CS, but struggled to see how this translated to everyday professional life, as the roles were seen as being intertwined. However, they understood that there was an internal logic between ES and CS, where CS was seen as the most important support structure, with practical advice and immediate feedback related to daily clinical work. In many cases, ES was only seen as relevant when there was concern about the resident’s performance or learning trajectory. Consequently, most supervisors did not see the need for scheduled conversations with well-functioning residents, and this was not prioritised in a busy clinical workplace with many competing tasks.

In this workplace setting, supervisors balanced multiple expectations and attempted to combine being a support person for residents with assessing their competencies. Providing professional and personal support and, at the same time, assessing residents’ performance has proven problematic for residents as well as challenging for educators, creating tension between feedback focused on growth and development and assessment focused on judgment and decision-making.^24^ If the purpose of the assessment is not made explicit, the learner is likely to feel the need to perform instead of revealing vulnerabilities, knowledge gaps, and potential problems, which in turn might hinder learning opportunities. Molloy and Bearman,^25^ used the term ‘intellectual candour’ to describe the relationship between revealing vulnerability on the one hand and appearing competent on the other. From the perspective of supervisors, implicit assessments are likely to make it more difficult to establish trusting relationships with trainees. Furthermore, given the time constraints and lack of support identified in our study, supervisors are under pressure to reconcile multiple unclear expectations. As shown in previous studies, clinicians with multiple educational roles and responsibilities may find it challenging to align personal and professional identities, values, and beliefs with their educational roles.^26^

Billett^9^ claimed that realising the full potential of workplace learning requires thorough preparation and careful staging. The way individuals engage in work practices has an impact on what and how they learn, where supervisors are important facilitators of residents’ learning. There seems to be a significant gap between ideals and reality in clinical practice when it comes to how ES is prescribed and how the workplace setting enables ES. Norcini and Zaidi^27^ argued that routine interactions among members of healthcare teams and between patients and trainees form the basis for assessment, and that this information is readily available. A pivotal factor for the successful use of this information, allowing hospital consultants to function as both assessors and supporting supervisors, is educators who are trained in assessment methods based on the observation of routine encounters. These methods can support the educational process by allowing feedback opportunities and, in turn, inform plans for remediation, if needed.

Supervisors assume great responsibility to ensure that residents are offered ES, even if finding time to do so comes at a personal cost. We identified multiple resources used by supervisors to enable ES. Guidelines recommend that supervision be scheduled in structured meetings, and supervisory relationships are assumed to strongly affect the effectiveness of supervision.^23^ Time and working hours that allow residents and supervisors to work side by side were highlighted as prerequisites for feedback, based on observations and role modelling in this study. Supervision based on other types of input – such as assessment meetings, work summaries, journals, or residents’ own reflections – was seen as incomplete and even unreliable.

Given that the delineation between ES and CS was difficult to translate into a clinical setting, supervisors need assistance in embedding various supervisory roles in practice. The necessity for faculty development when implementing new educational roles as part of the transition to competency-based medical education (CBME) is well established.^28^^,^^29^ In our study, we found that supervisors felt left alone to find suitable ways to apply guidelines and theories of supervision, whereas their efforts were met with little organisational, cultural, or collegial support. Based on our findings, we suggest longitudinal faculty development that facilitates the sharing of experiences and peer mentoring. Interventions should offer help beyond mastering ES and include assisting educators in the alignment of multiple educational and professional roles. Finally, educator training should be accompanied by structural and organisational changes, allowing supervisors to supervise in line with guidelines and best practices.^30^

### Strengths and weaknesses

The findings of this study are based on rich data from supervisors working in somatic medical specialities including internal medicine, surgery, and anaesthesiology. Recruitment by snowballing may have had an impact on our material, depending on participants’ interests and biases. Threats to validity include sampling bias, as our participants may represent hospital consultants who are particularly interested in ES. However, physicians with a special interest in supervision could also be those who have reflected on CS and ES, and thus provide rich material. Furthermore, quotes were translated from Norwegian transcripts into English, which poses the risk of nuances in meaning being lost.

### Implications for future research

The results of this study can contribute to informing educational processes in clinical practice and to filling a knowledge gap in educational practices on ES, its relation to CS, and a new understanding of how and why these roles are ambiguous. Building on the current study on supervisors’ perceptions of their role in supporting residents’ learning, more studies are needed to understand how ES is conducted in various residency training systems and hospital settings. Observational studies and the exploration of potential differences between specialties and supervisory traditions within specialities are particularly important.

## Conclusions

In this study we explored how supervisors reflect upon the importance of educational supervision and consequently their own role in the residents’ training in hospitals, and what responsibilities they take during the residents’ training. Overall, supervisors described ES as an important support function. However, in everyday practice, the role of educational supervisors remains unclear. Supervisors balance multiple expectations in ensuring the quality of residents’ training and are responsible for patient safety. Establishing a good relationship between residents and supervisors was considered the key to well-functioning ES. Supervisors provide support and simultaneously assess residents’ competencies, a balancing act that challenges the establishment of trusting relationships with the residents. Finally, we found that being a supervisor incurs personal costs in terms of the absence of organisational resources. Hospitals consultants revealed that the role of educational supervisors was unclear despite formal expectation following the CBME reforms. However, many educational institutions and teaching hospitals seem to be insufficiently prepared, underestimating the need for educator training that not only outlines expectations and ideals but also recognises the organisational barriers that characterise their professional practice.

### Acknowledgements

We would like to thank the participants in this study for sharing their experiences in practise as educational supervisors.

### Conflict of Interest

The authors declare that they have no conflict of interest.

## Supplementary material

Supplementary file 1 Appendix. Interview guide
