# Supplementary material for: Educational supervisor’s perceptions of their role in supporting residents’ learning: a qualitative study
Source: Int J Med Educ. 2023 Nov 20;14:178–86. doi: 10.5116/ijme.6544.cf18 (PMC10693950; doi:10.5116/ijme.6544.cf18)
Supplement: Supplementary file 1 — Appendix. Interview guide [file ijme-14-178-S1.pdf]

Appendix

Interview guide

Topics	Questions
Background	• Can you tell us briefly about your background and what you are working on?<br>• What experience do you have with training specialist candidates in your workplace?
Learning situations and support mechanism	• What type of training do residents need?<br>• Do you have any forums to talk about or plan follow-up of residents or supervision?<br>• Are there other important support mechanisms in learning situations that you think you can contribute to as a supervisor?
Perceptions of the supervisory role	• How would you define educational supervision to an outsider?<br>• What is your role in supporting residents training?<br>• What does an educational supervision session look like for you?
Previous experience with educational supervision	• How were you followed up in your resident training?
Importance of educational supervision in residents training	• What significance, in specialist education, do you think educational supervision has for learning?
